# Observation Unit Management of Older Adults with Rib Fractures: A Case Series

**DOI:** 10.5811/cpcem.62123

**Published:** 2026-08-31

**Authors:** Phillip D Jenkins, Elizabeth K Crawford Rowe, Colin Gershon, Mackenzie R Cook, John Marshall

**Affiliations:** * Oregon Health & Science University, Department of Surgery, Portland, Oregon; † Oregon Health & Science University, Department of Emergency Medicine, Portland, Oregon

**Keywords:** rib fractures, observation units, quality improvement, hospital resource utilization, older adults

## Abstract

**Introduction:**

Rib fractures in older adults carry high morbidity and mortality, leading many hospitals to admit patients based on age alone. The Rib Injury Guidelines (RIG) offer a triage system; under RIG 2, patients are typically admitted to inpatient care.

**Case Series:**

This study evaluated whether older adults categorized as RIG 2 solely due to age could be safely managed in an emergency department observation unit. We conducted a 14-month case series at an academic trauma center, reviewing 23 patients 60 years and older with rib fractures. Twenty met RIG 2 criteria, including those later admitted for nontrauma reasons. The mean observation stay was 26.6 hours, and the total hospital stay was 1.8 days. Seventeen patients (85%) were discharged directly from observation, three (15%) were admitted to medicine, and none returned within 72 hours.

**Conclusion:**

Carefully selected older adults with rib fractures may be safely managed in observation units, optimizing resource utilization and patient safety.

## INTRODUCTION

Rib fractures are common thoracic injuries in older adults and are associated with significant morbidity and mortality.^1^,^2^ Compared with younger patients, older adults suffer disproportionately from rib fracture complications, including pneumonia, respiratory failure, and death.^1^ Each additional rib fractured in a geriatric patient increases pneumonia risk by approximately 27% and mortality by nearly 20%.^1^,^2^ After adjusting for comorbidities and injury severity, trauma patients 65 years of age and older have five-fold higher odds of death from chest injuries compared with younger counterparts.^3^ These risks make accurate triage and timely management a priority for improving quality and outcomes in this vulnerable population. Traditional risk stratification, using age and the number of rib fractures, was based on the 2001 Bulger study. As imaging has improved over the past quarter-century, it has become clear that risk stratification that accounts for pain/respiratory function could lead to more appropriate resource use.^1^

Although traditional teaching has favored intensive care unit (ICU) admission for elderly rib fracture patients, structured management protocols now guide disposition decisions, helping clinicians identify which patients are safe for discharge and which need only a standard ward bed.^1^ The Rib Injury Guidelines (RIG) provide a standardized approach for triage based on age, number of rib fractures, comorbidities, pain control, pulmonary function, and other risk factors.^4^ (Table 1). Patients meeting RIG 1 criteria are generally safe for discharge, RIG 2 patients typically require inpatient admission, and RIG 3 patients are recommended for ICU-level monitoring. In a prospective validation study, RIG implementation reduced unnecessary ICU admissions and shortened length of stay without compromising safety.^5^ Other rib fracture protocols have similarly demonstrated improved efficiency and outcomes when applied to older trauma patients.^6^

Despite these advances, practice variation remains substantial. In a study of nearly 24,000 patients 65 years of age and older with isolated rib fractures, ICU admission rates varied from 0% to 92% across trauma centers, with higher utilization centers demonstrating lower pneumonia and complication rates.^7^ Such findings highlight the tension between aggressive monitoring and resource stewardship. While guidelines recommend admission for many older adults, some patients, particularly those whose only RIG 2 criterion is age, may not require a full hospital admission if appropriately monitored.

Emergency department observation units (EDOU) represent a significant opportunity for optimizing the management of blunt chest trauma patients, often reducing hospital admissions and length of stay (LOS) without compromising safety.^6^ Prior studies affirm that carefully selected patients can be safely managed in observation settings, leading to positive clinical and financial outcomes and RIG protocol provide us a validated structure to carefully select those patients.^7^ The application of EDOUs aligns with previous work estimating a national savings of approximately $3.1 billion annually,^8^ underlining the financial benefit of using observation units for short-stay patients, such as those with less severe blunt chest trauma who do not require extensive inpatient care.

Moreover, studies specifically addressing trauma patients further validate the efficacy of observation units. One study highlighted that blunt chest trauma constitutes a significant portion of trauma presentations in EDs, and identifying which patients require intensive monitoring can be challenging.^9^ This analysis suggests that certain subsets of these patients can be effectively managed on a less intensive basis, which resonates with the operational framework of EDOUs. By implementing a selective observation strategy, hospitals may observe a decrease in LOS and a likely reduction in related complications such as hospital-acquired pneumonia, as noted in research linking inadequate pain control with extended admissions in chest trauma cases.^10^

Our study evaluates a targeted application of RIG 2 triage to older adults whose only qualifying factor was age. We present a case series of patients managed in an EDOU rather than admitted to the hospital, to assess feasibility, safety, and resource use. By integrating guideline-based triage into observation unit practice, this work provides proof of concept for a novel approach to rib fracture management and a foundation for future studies.

*CPC-EM Capsule*

What do we already know about this clinical entity?

*Rib fractures in older adults carry high morbidity. Rib Injury Guidelines (RIG) 2 patients are typically admitted to an inpatient ward, often based on age alone.*

What makes this presentation of disease reportable?

*This is the first report of managing age-only RIG 2 patients in an emergency department observation unit (EDOU), demonstrating shorter stays without 72-hour returns.*

What is the major learning point?

*Older adults meeting RIG 2 criteria solely due to age may be safely managed in an EDOU, reducing unnecessary inpatient admissions.*

How might this improve emergency medicine practice?

*Integrating guideline-based triage with EDOU pathways can reduce inpatient admissions, lower costs, and preserve safety for selected older rib fracture patients.*

## METHODS

### Study Design and Setting

This was a case series conducted at an academic Level I trauma center over a 14-month period from February 2023 to April 2024. The trauma and ED teams jointly decided to incorporate the RIG scoring tool into hospital policy to help guide the disposition of patients with rib fractures. Following this initial implementation, a second plan-do-study-act cycle was conducted in early 2024 to evaluate outcomes across various patient groups with rib fractures. This review identified well-appearing older patients categorized as RIG 2 only due to their age as a potentially safe cohort for EDOU admission, ultimately leading to the 14-month trial on which this paper is based. As a result of this second plan-do-study-act cycle, modified RIG protocol was implemented to guide disposition decisions for older adults with rib fractures. Under this protocol, patients were managed in the EDOU when their clinical condition permitted, rather than being routinely admitted to the hospital. At our institution, “triage” in this context refers to the disposition decision made by the trauma and emergency medicine teams after initial ED evaluation, not the conventional acuity-based triage that occurs at ED arrival. After workup, eligible patients were assigned to EDOU-level care under the modified RIG pathway. A subset of these patients subsequently required inpatient admission to the hospital medicine service for nontrauma medical reasons (eg, management of comorbid conditions identified during observation); these patients were retained in the analytic cohort because they met all inclusion criteria at the time of EDOU assignment.

### Inclusion and Exclusion Criteria

All patients 60 years of age and older with rib fractures who were triaged to the EDOU during the 14-month study period were eligible for review. Patients were included in the analytic cohort if they were managed initially in the EDOU and had a reviewer-calculated RIG score of 4 points, with points for age 60 and older only. Patients who were admitted to an inpatient service after an initial EDOU stay for nontrauma medical reasons were retained in the analytic cohort, as they met all inclusion criteria at the time of triage.

Patients were excluded if they were not actually managed in the EDOU during the index visit. Specifically, two patients were excluded because they were admitted directly to a hospital-based observation unit rather than the EDOU, and one patient was excluded because their rib fractures occurred during a different encounter outside the study window.

### Data Collection and Scoring

Electronic health records were reviewed. One author retrospectively reviewed RIG scores based on available chart data, including demographics, comorbidities, injury details, and hospital course. Emergency department observation unit LOS, total hospital LOS, Injury Severity Score (ISS), comorbidities, and 72-hour return visits were extracted for analysis.

### Analysis

We used descriptive statistics to summarize patient demographics, injury characteristics, LOS, comorbidities, and return visits. Continuous variables are reported as mean (standard deviation) and median with interquartile range (IQR). Categorical variables are reported as counts and percentages.

### Ethics

This study was reviewed and approved by the institutional review board (STUDY00027037).

### Statistical Software

All analyses were performed in Python version 3.12.4 (Python Software Foundation,). Data management and descriptive statistics were conducted using pandas, NumPy, and SciPy. Tables were prepared with python-docx.

## RESULTS

A total of 23 patients 60 years of age and older with rib fractures were triaged to the EDOU during the study period. Three patients were excluded from analysis: two were admitted directly to hospital-based observation units rather than the EDOU; and one had rib fractures at a separate encounter outside the study window. The final analytic cohort included 20 patients managed initially in the EDOU.

### Analytic Cohort (n = 20)

Patients had a mean age of 73.7 years (SD 9.2, median 71.5, IQR 65.5–83.0) and a mean ISS of 9.2 (SD 3.6, median 10, IQR 7.3–10.3). Observation unit LOS averaged 26.6 hours (SD 15.4, median 21.1, IQR 14.6–41.0). Total hospital LOS averaged 1.8 days (SD 1.2, median 1.3, IQR 0.9–2.2). No patients returned to the ED within 72 hours. Comorbid conditions were common, present in 18 patients (90%). A summary of patient characteristics is provided in Table 2.

## DISCUSSION

This case series demonstrates that a structured rib fracture triage protocol may be adapted to safely manage selected older adults in an EDOU. Specifically, patients whose RIG 2 categorization was based solely on age were observed for short periods, discharged without subsequent admissions, and had no 72-hour return visits. This is a novel approach not described in the original RIG studies and is being implemented through a thoughtful, rigorous plan-do-study-act cycle. By aligning guideline-based triage with observation unit resources, this approach reduced inpatient admissions while preserving patient safety.

Historically, older adults with rib fractures have been admitted reflexively due to age-associated risk, despite heterogeneity in their actual clinical needs. By separating age-only RIG 2 patients from those with additional risk factors, hospitals may reduce unnecessary admissions while still identifying patients who require higher. intensity monitoring. The result is a more precise allocation of hospital resources and a care model that balances safety with stewardship.

From a cost perspective, we know that the elderly population in the United States is growing significantly, such that recent estimates place one in five individuals being 65 years or older by 2030, and contributes significantly to rising inpatient trauma costs.^11^ Review of Centers for Medicare & Medicaid Services data review shows that in patients admitted for similar diagnoses, short inpatient stays cost approximately three times that of observation stays (~US $5,142 for a short inpatient stay versus ~US $1,741 for an observation stay).^12^ The potential to manage appropriately risk-stratified patients in an EDOU could significantly decrease the cost of appropriate care in this population.

Our results also highlight the importance of differentiating rib fracture–related care needs from nontrauma medical drivers of admission. Patients excluded from the analytic cohort were hospitalized for comorbid or unrelated medical conditions, underscoring that outcomes depend not only on injury severity but also on broader clinical context. This distinction is critical for hospitals seeking to reduce LOS, prevent avoidable admissions, and ensure comprehensive care for medically complex older adults.

## LIMITATIONS

This study is limited by its small sample size and single-center design, which may affect generalizability. The absence of a direct inpatient comparator group limits conclusions about relative outcomes. Finally, observation units vary in availability and structure across healthcare systems, which may influence the feasibility of adopting this approach in other institutions.

## CONCLUSION

A modification of the Rib Injury Guidelines demonstrated that older adults with rib fractures categorized as RIG 2 based on age alone may be safely managed in an EDOU setting. This strategy reduced inpatient admissions and overall length of stay without compromising safety. Larger multicenter studies with comparator groups are needed to confirm these findings, refine selection criteria for age-only RIG 2 patients, and evaluate downstream outcomes such as readmission, functional recovery, and cost. This case series provides a foundation for those investigations and supports continued protocol development as observation unit pathways for older adults with rib fractures mature.

## Figures and Tables

**Table 1:** Rib Injury Guidelines (RIG) classification system used in a study that evaluated whether older adults categorized as RIG 2 solely due to age could be safely managed in an observation unit.

Category*	Recommended disposition	Representative criteria
RIG 1 (low risk)	Discharge home	Younger age, few rib fractures, adequate pain control, preserved pulmonary function and cough, no significant comorbidities, and no high-risk imaging features
RIG 2 (moderate risk)	Inpatient ward admission	Older age (≥ 60 years), moderate number of rib fractures, mild-to-moderate comorbidities, pain partially controlled, mildly impaired pulmonary function or cough, or other intermediate risk features not meeting RIG 3 criteria
RIG 3 (high risk)	ICU-level monitoring	Multiple rib fractures, bilateral fractures, flail chest, pulmonary contusion, significant comorbidities (eg, COPD), poorly controlled pain, impaired cough, or markedly reduced inspiratory spirometry

* Categories are adapted from Nelson et al.^4^

*COPD*, chronic obstructive pulmonary disease; *ICU*, intensive care unit; *RIG*, Rib Injury Guidelines.

**Table 2:** Characteristics of patients ≥ 60 years triaged to emergency department observation with rib fractures.

Variable	RIG 2 (0 if not for age) (n = 20)
Age (years)	73.7 (9.2) (Median 71.5, IQR 65.5–83.0)
ISS	9.2 (3.6) (Median 10, IQR 7.3–10.3)
EDOU LOS (hours)	26.6 (15.4) (Median 21.1, IQR 14.6–41.0)
Total LOS (days)	1.8 (1.2) (Median 1.3, IQR 0.9–2.2)
Return ≤ 72 hours	0 (0%)
Comorbidities	18 (90%)
Admitted from EDOU for Trauma	0 (0%)

*EDOU*, emergency department observation unit; *IQR*, interquartile range; *ISS*, Injury Severity Score; *LOS*, length of stay; *RIG*, Rib Injury Guidelines.
